# Population genomics and evidence of clonal replacement of *Plasmodium falciparum* in the Peruvian Amazon

**DOI:** 10.1038/s41598-021-00806-5

**Published:** 2021-10-27

**Authors:** Fredy E. Villena, Stephen E. Lizewski, Christie A. Joya, Hugo O. Valdivia

**Affiliations:** 1 U.S. Naval Medical Research Unit No. 6 (NAMRU-6), Lima, Peru; 2 Facultad de Ciencias Biológicas, Universidad Nacional Mayor de San Marcos, Lima, Peru; 3 Vysnova, Lima, Peru

**Keywords:** Parasite genomics, Genomics

## Abstract

Previous studies have shown that *P. falciparum* parasites in South America have undergone population bottlenecks resulting in clonal lineages that are differentially distributed and that have been responsible for several outbreaks different endemic regions. In this study, we explored the genomic profile of 18 *P. falciparum* samples collected in the Peruvian Amazon Basin (Loreto) and 6 from the Peruvian North Coast (Tumbes). Our results showed the presence of three subpopulations that matched previously typed lineages in Peru: Bv1 (n = 17), Clonet D (n = 4) and Acre-Loreto type (n = 3). Gene coverage analysis showed that none of the Bv1 samples presented coverage for *pfhrp2* and *pfhrp3*. Genotyping of drug resistance markers showed a high prevalence of Chloroquine resistance mutations S1034C/N1042D/D1246Y in *pfmdr1* (62.5%) and K45T in *pfcrt* (87.5%). Mutations associated with sulfadoxine and pyrimethamine treatment failure were found on 88.8% of the Bv1 samples which were triple mutants for *pfdhfr* (50R/51I/108N) and *pfdhps* (437G/540E/581G). Analysis of the *pfS47* gene that allows *P. falciparum* to evade mosquito immune responses showed that the Bv1 lineage presented one *pfS47* haplotype exclusive to Loreto and another haplotype that was present in both Loreto and Tumbes. Furthermore, a possible expansion of Bv1 was detected since 2011 in Loreto. This replacement could be a result of the high prevalence of CQ resistance polymorphisms in Bv1, which could have provided a selective advantage to the indirect selection pressures driven by the use of CQ for *P. vivax* treatment.

## Introduction

Malaria is an infectious disease caused by parasites of the *Plasmodium* genus that are transmitted through the bite of infected anopheline mosquitoes. This disease is widespread in tropical and subtropical regions causing more than 228 million cases and 405,000 deaths in 2018^1^. The region of the Americas reported 929,000 cases and 577 deaths in 2018 with a population at risk of 138 million in 19 countries^1^.

Recently, South America has experienced a rise in malaria cases due to gold mining activities and increased transmission in Venezuela^2,3^. More than 90% of all malaria cases in the region were reported in Venezuela (51%), Brazil (23%), Colombia (10%) and Peru (6%)^1^.

In Peru, more than 95% of all malaria cases come from the North Eastern Amazon (Loreto region). In this region, *Plasmodium vivax* is more prevalent than *P. falciparum* at a ratio of 4:1^4^. Malaria transmission is perennial in the country and the majority of affected people perform activities or live near rivers or natural water bodies^5,6^. Other malaria endemic areas are the Southern Amazon (Madre de Dios region) which is affected by illegal gold mining and the Pacific North Coast (Tumbes and Piura regions) where malaria transmission is intermittent^7^.

Although *P. vivax* has historically been prevalent in Peru, *P. falciparum* has expanded to different geographical areas. Until 1988, *P. falciparum* cases were only reported on the borders of Peru with Ecuador, Colombia and Brazil^8^. However, in 1991, 140 cases of *P. falciparum* were reported in Loreto and by 1994 the first cases were detected in Iquitos, the capital of the Loreto region and infections increased up to 54,290 cases in 1997^8^. This expansion of cases was associated with the introduction of *Anopheles darlingi*, a highly competent anthropophagic vector, to Iquitos in 1991 and a rapid increase in abundance by 1994^4,8,9^. Currently, *An. darlingi* and *An. benarrochi* are widely distributed in the Amazon region whereas *An. albimanus* is the prevalent vector in the North Coast^10,11^.

Another aspect that has characterized Loreto is the rapid emergence of drug resistant *P. falciparum* strains to chloroquine and sulfadoxine pyrimethamine^12,13^. In the early 1990’s, the first drug resistant cases were reported in Peru; chloroquine (CQ) resistance in the north coast and CQ and sulfadoxine-pyrimethamine (SP) in the Amazon region^12^. These results were used to implement SP as a first-line therapy to *P. falciparum* in the North Coast and demonstrated the presence of two different ecosystems in Peru. In 2001, Peruvian Government implemented a new malaria treatment scheme for *P. falciparum* based on artemisinin combination therapies (ACTs); Mefloquine-artesunate (AS) in the Amazon and AS-SP in the North Coast^12,14^.

Studies suggested the presence of three circulating *P. falciparum* lineages in Peru; two exclusively circulating in Loreto and a mixed lineage in both Loreto and the north coast^15,16^ . However, after the peak of malaria resurgence in Peru (between 1999 and 2000), molecular studies showed five circulating clonets (A, B, C, D and E) in the Peruvian territory^15^. All clonets were circulating in the Amazon region but only one in the North Coast (clonet E). Biodiversity analysis suggested that clonet E suffered a bottleneck and that only clonet B had evidence of rapid expansion. A subsequent analyses with samples from 2006–2007 showed evidence for the admixture of existing clonets due to outcrossing and appearance of new hybrid recombinants clonets such as B/C and C/D^15^.

In 2010–2012, one *P. falciparum* outbreak occurred in the North Coast of Peru five years after the elimination of malaria (no local *P. falciparum* transmission for 5 years was reported) in that region^17^. Epidemiological research showed that two of the first cases from this outbreak were from military personnel who were previously deployed in the Peruvian Amazon. Molecular typing showed that the North Coast samples were closely related but not completely identical to clonet B and thus were named as clonet B variant 1 (Bv1). This clonet variant presented point mutations in several drug resistance associated genes including *pfdhfr, pfdhps, pfcrt* and *pfmdr1*, as well as deletions on *pfhrp2*^17^.

In 2013, another outbreak produced by *P. falciparum* Bv1 was reported in the region Cusco Region (southern of Peru) that did not present *P. falciparum* cases since 1946^18^. A study conducted on samples from this outbreak suggested a potential introduction of Bv1 from the Peruvian Amazon^18^.

These outbreaks in distinct regions underscore the need for continuing surveillance of circulating parasites in Loreto, a region where *P. falciparum* lineages can spread to the rest of the country by human migration. In this study, we explored potential changes in circulating *P. falciparum* strains in Loreto in order to assess if the Bv1 clonet has replaced other *P. falciparum* circulating lineages in the Peruvian Amazon Basin and explore genotypic variants associated with drug resistance and virulence factors. The information provided by this study will serve to support control programs, current treatment regimens and regional malaria eradication efforts.

## Methods

### Study design

**Figure 1:**
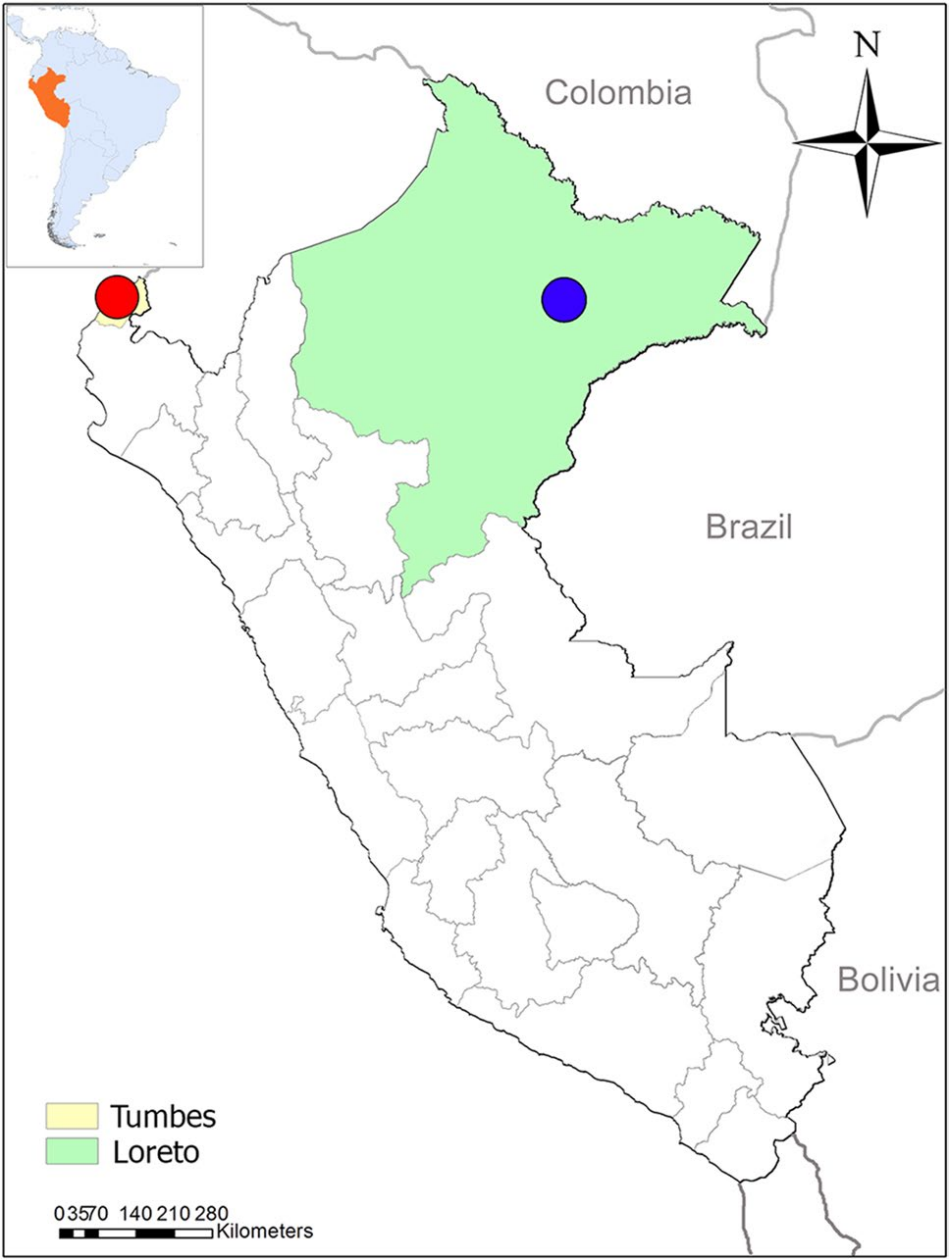
Collection sites. Samples for this study came from *P. falciparum* specimens collected in the Peruvian North Coast (red dot) and Amazon Basin (blue bot) between 2006 and 2017. The map was created using ArcGIS online (ESRI Inc. Redlands, CA, USA. https://www.esri.com/) using open data obtained from GADM database of Global Administrative Areas, version 3.6. www.gadm.org.

Samples were collected from the Peruvian Regions of Tumbes in the North Coast and Loreto in the Northern Amazon Basin (Fig. 1). Samples from Tumbes were collected between 2010 and 2011 whereas samples from Loreto were collected between 2006 until 2017.

Loreto presents a tropical climate with the rainy season from November to May and the dry season between June and October. Annual average temperature is 27 °C with more 80% humidity and average annual rainfall of 4 m.

Tumbes presents a tropical climate with the rainy season from January to April and the dry season between June and November. Annual average temperature is around 25 °C, 60% humidity and average annual rainfall of 175 mm.

### Ethics

The samples tested for this study were selected from two sources: (i) a passive surveillance study approved by the Institutional Review Board of the U.S Naval Medical Research Unit 6 (NAMRU-6) in compliance with all applicable federal regulations governing the protection of human subjects (protocol NMRCD.2007.0004) and (ii) a secondary data analysis with samples collected under outbreak and public health activities (NAMRU6.2018.0002) which did not involve humans as the subjects of the study evaluation. Informed consent was obtained from all participants and/or their legal guardians for study NMRCD.2007.0004. Informed consent was not required for NAMRU6.2018.0002 since it does not meet the definition of research involving human subjects per US Code of Federal Regulations, 32 CFR Part 219—PROTECTION OF HUMAN SUBJECTS, Section 219.104 Exempt research.

### Study procedures

Blood samples were collected from suspected malaria patients and used to prepare slides for malaria diagnosis by microscopy. Positive samples were stored at −20 °C and then sent to NAMRU-6 Lima for DNA extraction and molecular testing. DNA was extracted from 200ul of whole blood using the QIAamp® DNA Blood Mini Kit (GmbH, Hilden, Germany) following the manufacturers protocol and stored at −20 °C. *P. falciparum* monoinfection was confirmed by a nested PCR that targets 18S small subunit ribosomal RNA (ssrRNA) gene^19^.

### Selective whole genome amplification

Monoinfection samples were sequenced using whole genome amplification as previously described^20^. Briefly, we performed two amplification rounds with 50 ng of input DNA to a 50ul reaction mixture containing 5 μM SWGA primers (set 6A and 8A). Each amplification mix contained 30U phi29 DNA polymerase enzyme (New England Biolabs), phi29 DNA buffer (New England Biolabs), 1% bovine serum albumin, 1 mM dNTPs and water. The amplification reaction was carried out on a thermocycler and consisted of a ramp down from 35 to 30 °C during 10 min per degree, 16 h at 30 °C, 10 min at 65 °C and hold at 4 °C.

Amplified samples were diluted in 1:1 molecular grade water and purified using the Ampure XP (Beckman-Coulter) beads system at a ratio of 1:1 as per manufacturer’s protocol. Paired-end sequencing libraries were generated using 1 ng amplified DNA using the Nextera XT kit (Illumina, California, USA) following the manufacturer’s protocol. The pooled library was sequenced using the MiSeq reagent kit v3 (600 cycles) and MiSeq reagent kit v2 (500 cycles) and sequenced on an Illumina MiSeq sequencer to generate 250–300 bp paired end reads.

### Read mapping and variant calling

Reads were quality filtered using Trimmomatic v0.36 with minimum base quality cutoff of 20, leading and trailing base qualities of 20, minimum per base average quality of 20 and a minimum read length of 70 bp^21^.

Filtered reads were competitively mapped onto the *P. falciparum* 3D7 (v33)^22^ and human reference (GRCh38.p13)^23^ genomes using Bowtie2 v2.3.5^24^. Read depth information was used to estimate coverage for *pfhrp2* and *pfhrp3* for each sample.

Single nucleotide variants were jointly called from all samples using GATK v4.1.4.0 under the haplotype caller module and following the GATK best practices for variant calling^25^. Called variants were filtered using a mapping quality score greater than 40.0 and quality by depth greater than 2.0. SNP density was visualized with the BioCircos package implemented in R^26^. Genes located in highly polymorphic regions were extracted and used for gene ontology enrichment analysis on PlasmoDB^27^ using Bonferroni for correction for multiple testing to control significant results. Next generation sequencing data on drug resistance markers was complemented with Sanger sequencing results that were previously obtained^17^.

### Analysis of diversity and selection

In order to assess the overall diversity at the individual and population level, we employed the FWS metric as previously described^28^. For this purpose, we called SNPs with SAMtools^28^ and employed moimix (https://github.com/bahlolab/moimix) to calculate the corresponding within-host heterozygosity (FWS).

Population scaled Tajima’s D was evaluated using a sliding window of 5 kb. Regions under selection were selected and the corresponding genes retrieved for gene ontology enrichment analysis using Bonferroni correction in PlasmoDB^27^.

In order to explore polymorphisms that could be associated with drug resistance, we employed the SnpEff tool^30^ to analyze the effects of SNPs in reported drug resistance genes.

### Population structure and phylogenetic analyses

Filtered SNPs were subsequently used for discriminant analysis of principal components using the adegenet package implemented in R^31^. In order to provide additional information regarding the parasite population structure in these foci we used Admixture for estimating individual ancestries^32^. Admixture analyses were carried out using 10 independent Markov Chain Monte Carlo runs for each k of 1 to 10 under a tenfold cross-validation procedure with 1000 pseudoreplicates using different initial seed values for each K. We used Admixture’s cross-validation to select the most likely value of K and then employed pophelper to generate multiline plots^33^.

Filtered SNPs were used to assess the phylogenetic relationship of the isolates collected in Loreto and Tumbes. For this purpose, we generated a multiple sequence alignment in Seaview^34^ and phylogenetic reconstruction under a maximum likelihood approach in PhyML using the general time reversible model and 1000 pseudoreplicates^35^. Single nucleotides polymorphism of the S47 gene were individually analyzed by an haplotype network created in PopArt 1.7^36^. In addition, relatedness between sample pairs was assessed by estimating the pairwise identity by descent (IBD) using hmmIBD with 100 iterations^37^. Samples were classified according to their IBD as highly-related (25–49%), very highly-related (50–75%) and clonal (> 75%). IBD networks were created according to time and collection site on cytoscape^38^.

## Results

### Sample collection and whole genome sequencing

Blood samples were collected from 24 patients with *P. falciparum* monoinfection as determined by nested-PCR. Out of those, six samples were from the North Coast (collected during the outbreak in 2010–2011) and 18 from the Northern Amazon Basin (nine from 2006 to 2011 and nine from 2012 to 2017).

SWGA resulted in 24 high-quality *P. falciparum* whole genome sequences with an average of 2,492,353 reads per sample. Competitive read alignment against the *P. falciparum* 3D7 and human reference genomes resulted in an average 94.6% (SD: 4.7%) of reads aligning to the *P. falciparum* genome. The average read depth was of 20.47× (SD: 8.78×) with 52.43% (SD: 15.65%) of the nuclear genome covered by ≥ 5 reads (Table S1).

### Chromosome SNP density

**Figure 2:**
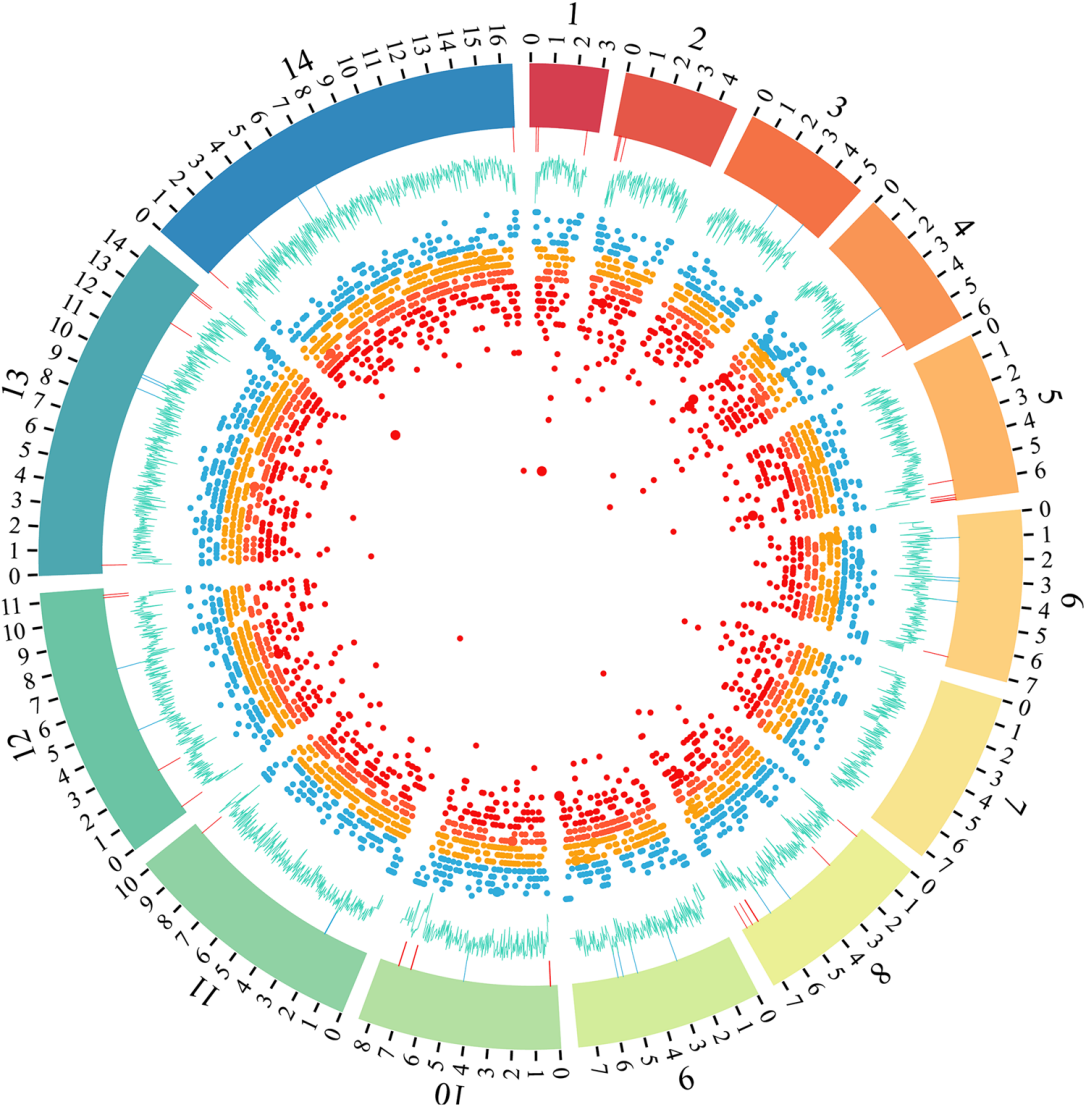
Genomic wide scans of SNP density and Tajima’s D. The figure shows SNP density in quartiles (blue, orange, red and dark red) across the 14 *P. falciparum* chromosomes (internal dot plot) showing regions with high density on chromosomes 1, 2, 4, 5, 7, 8, 11, 13 and 14. Population scaled Tajima’s D shows regions under positive selection on chromosomes 1–6, 8, 10–14 (external blue line plot). Coordinates of genes under positive (red) or negative (blue) selection are shown as lines after the Tajimas’D line plot.

A total of 38,321 single nucleotide polymorphisms (SNPs) were called across all samples. Outlier regions with high SNP density were detected on chromosomes 1, 2, 4, 5, 7, 8, 11, 13 and 14 (Fig. 2). Gene enrichment analysis showed significant enrichment for molecular functions associated to host cell surface binding (Bonferroni-corrected *p* < 0.05) (Table S2).

Genomic wide scan using 5 kb sliding windows showed a tendency towards neutrality (mean Tajima's D = −0.5) (Fig. 2) with 61 chromosomic regions under selection (Table S3). All chromosomes but one (chromosome 7) presented a region under selection. Chromosomes 1, 2 and 3 only presented regions under positive selection whereas chromosomes 3 and 9 only under negative selection.

Regions under positive selection included genes members of the immunogenic variant surface antigens (*rifin*, *stevor* and EMP1) that have important roles in pathogenesis and immune evasion^39^. This information suggests that selective pressure on these parasites may lead to positive selection on diversity maintaining the heterozygosity pool in the population.

SNPEff showed that 0.11% of the variants had a high impact on their corresponding genes related to loss of function or protein truncation, 15.02% had a moderate impact associated with non-disruptive variant that might affect protein functionality, 6.73% presented a low impact (synonymous variant) and 78.12% modifier impact associated with non-coding variants or variants affecting non-coding genes (Table S4). Also, 184,692 transitions and 160,006 transversions were identified (Ts/Tv ratio: 1.15).

Gene ontology enrichment analysis showed statistical significance enrichment on genes suffering from moderate impact variants. Enrichment was associated with host cell surface binding, motor activity, sulfur compound binding, calmodulin binding and actin binding (Bonferroni-corrected *p* < 0.05) (Table S2).

The majority of samples collected from both study sites were monoclonal with only one polyclonal sample (FWS < 0.95) that was collected from the Peruvian Amazon Basin.

### Drug resistance markers diversity

We identified widespread mutations associated with CQ and SP resistance in most isolates of the Peruvian Amazon Basin and North Coast (Table 1).

**Table 1:**
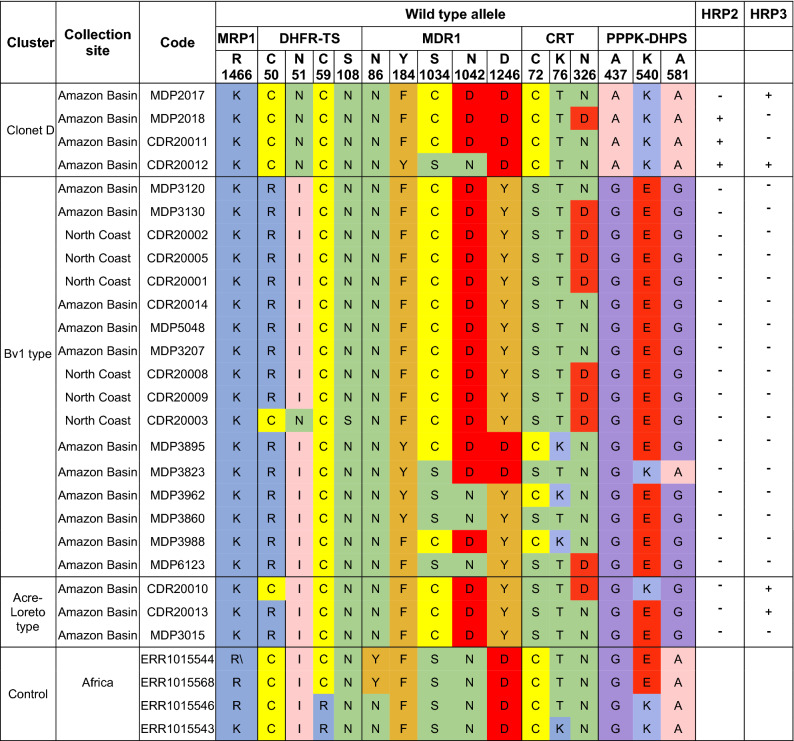
Multilocus drug resistance haplotypes.

The table shows SNPs on drug resistance genes on Peruvian and control African samples and *pfhrp2 and pfhrp3* presence based on coverage. The table is divided into three clusters according to the subpopulation structure. Colors on the amino acids indicate amino acid change according to the Zappo color scheme (Aliphatic/hydrophobic in pink, aromatic in orange, positive in blue, negative in red, hydrophilic in green, conformational special in magenta and cysteine in yellow).

In *pfmdr1*, we found that 62.5% of the isolates (n = 15) presented the S1034C/N1042D/D1246Y triple mutation that is associated with CQ-resistance and increased susceptibility to mefloquine, halofantrine and artemisinin^40^. This genotype is enhanced by several mutations in the *pfcrt* gene including a change on exon two that is associated with CQ resistance (K76T)^41^.

The majority of Bv1-type samples (16 out of 18) were triple mutants for *pfdhfr* (50R/51I/108N) and *pfdhps* (437G/540E/581G). Such a combination of alleles is strongly associated to sulfadoxine and pyrimethamine treatment failure^42^.

We did not find mutations on positions 1226 of *pfmdr1*; 73, 74, 75 and 356 of *pfcrt;* 436 and 613 of *pfdhps* nor 59 and 164 of *pfdhfr* that have been previously associated with drug resistance^43,44^.

None of the Peruvian samples presented mutations on *pfk13, pfatg18, pfarps10* and *ferredoxin* that have been associated with artemisinin resistance^45,46^ nor *pfexo which is* associated with piperaquine^47^ nor *pfmrp1* which is associated with sulfadoxine/pyrimethamine and artemether/lumefantrine resistance^48^.

**Figure 3:**
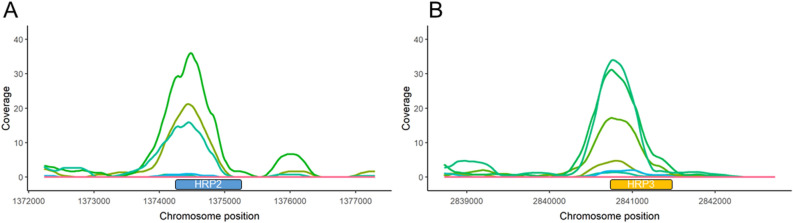
Read depth on *pfhrp2* and *pfhrp3* for all samples. (**A**) Three samples presented coverage for the *pfhrp2* gene. (**B**) Four samples presented coverage for the *pfhrp3* gene. None of the Bv1-type samples presented coverage on *pfhrp2* nor *pfhrp3*. The y-axis denote read depth whereas the x-axis indicates the nucleotide position in base pairs in chromosomes 8 (**A**) and 13 (**B**), respectively. *Pfhrp2* and *pfhrp3* gene positions are showed as boxes on the x-axis. In order to facilitate visualization, read depth was plotted using a sliding window of 500 bp and samples with coverage along the gene have been plotted as green shade colored lines.

Because of the high rates of *pfhrp2* and *pfhrp3* gene deletions in the Peruvian Amazon, we investigated the presence of these deletions in based on read depth. Our results show that none of the Bv1-type samples (n = 17) nor Acre-Loreto type (n = 3) presented coverage for *pfhrp2* in contrast to three Clonet D (75%) samples that presented coverage along the gene (Fig. 3). In the case of *pfhrp3*, none of the of the Bv1-type samples (n = 17) presented coverage for this gene whereas two Clonet D (50%) and two Acre-Loreto (66.6%) presented coverage along the gene. All samples with read depth for these genes were collected between 2006 and 2011.

### Population structure

**Figure 4:**
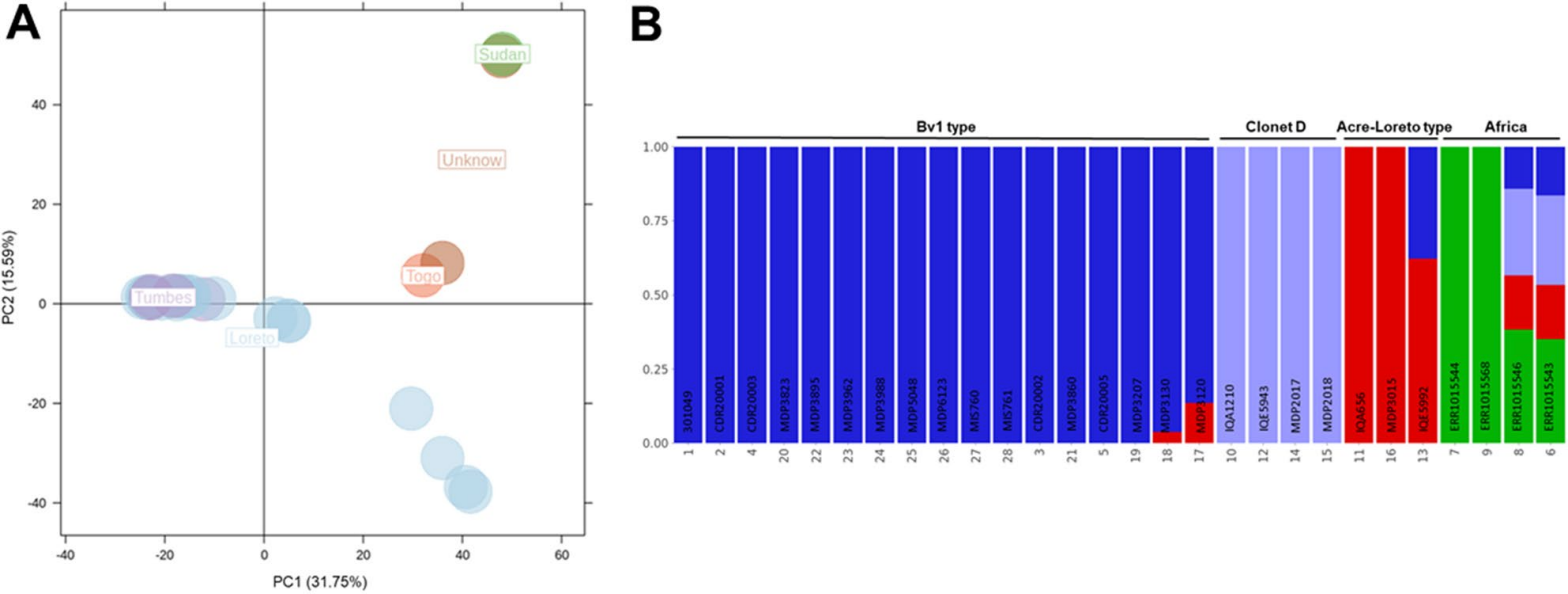
Population structure of *P. falciparum* samples. (**A**) Principal component analysis (PCA) showing three potential clusters for samples from Tumbes and Loreto. (**B**) Admixture analysis confirms the presence of these three Peruvian subpopulations, one of these (blue) that included all Bv1-type samples from the Peruvian Amazon Basin and North Coast regions and the other two corresponding to Clonet D and Acre-Loreto type, respectively. African reference samples were included as controls.

Principal component analysis and admixture revealed the presence of three parasite sub-populations and evidence of recombination between them in Peru (Fig. 4A and B). In the case of PCA, 47.3% of the variance was explained by the first two components. Admixture cross-validation for each clustering k from 1 to 10 showed that clustering with k = 4 presented the highest Delta K value (Figure S1 thus yielding three subpopulations in Peru.

The separation by PCA and admixture also showed three clusters in Peru. One consisting of a mixture of samples from the North Coast and the Amazon Basin (Bv1-type samples) whereas the other two clusters presented samples exclusively from the Amazon Basin (Clonet D and Acre-Loreto type) (Fig. 4B). This finding is supported by previous studies in Peru that indicate that the *P. falciparum* population is organized into up to 5 different clonal lineages which seem to be widely spread in the country with the highest level of admixture in the regions near Iquitos city^15,17,18^.

**Figure 5:**
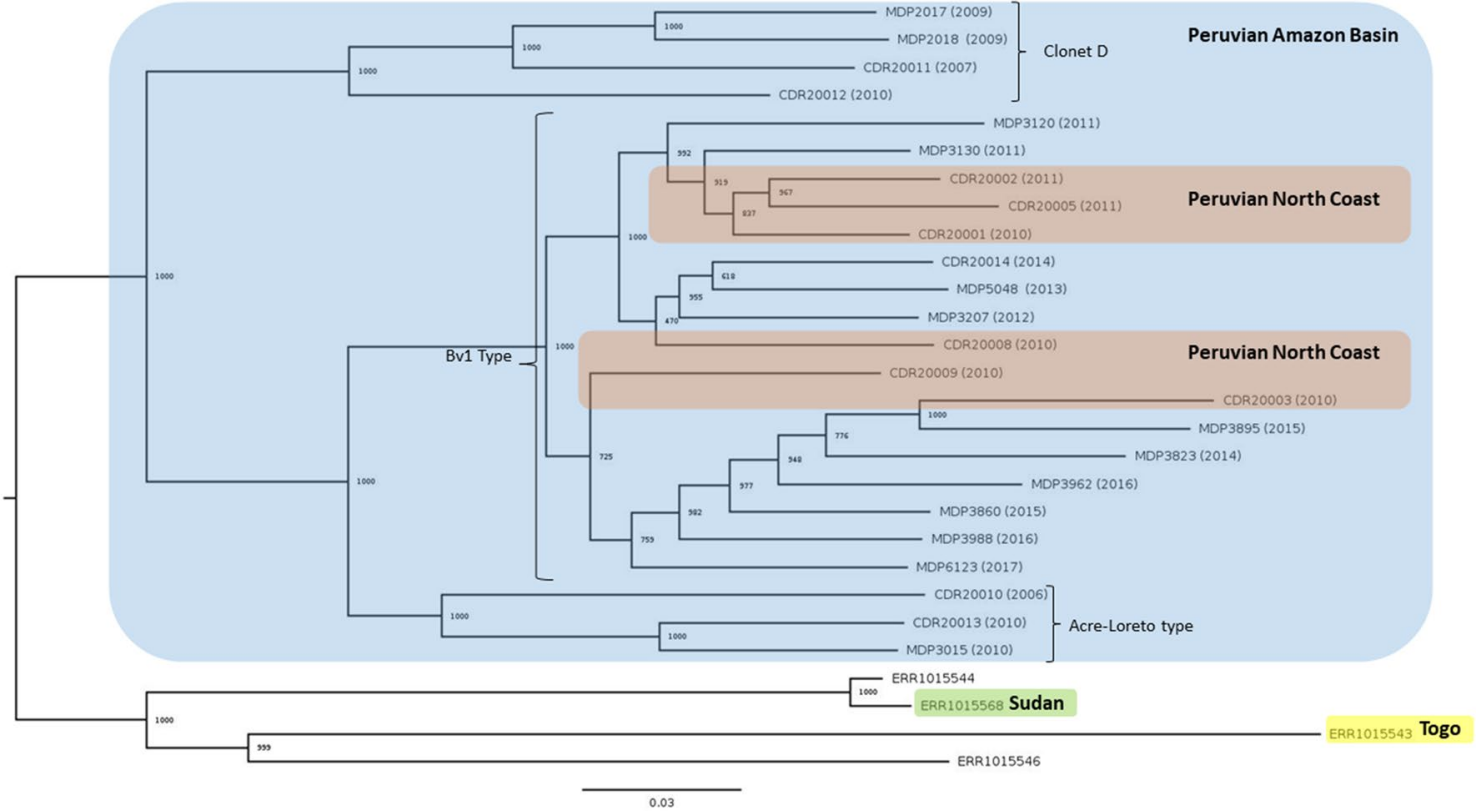
Maximum likelihood phylogenetic analysis. The phylogenetic tree shows the presence of three subpopulations from which only one (Bv1-type) was exclusively isolated since 2011 in the Peruvian Amazon Basin suggesting a population replacement in that region. Most of the branches of the tree are strongly supported by bootstrap values equal to 1000.

Whole genome maximum likelihood phylogenetic analysis matched the result from PCA and admixture showing that all samples collected since 2011 in the Peruvian Amazon Basin corresponded to the Bv1-type lineage (Fig. 5).

IBD analysis showed 80.5% of related sample pairs (IBD ≥ 25%) with 54.8% of them classified as highly-related pairs (IBD 25–49%), 22.9% as very highly-related (IBD 50–75%) and 2.7% as clonal (IBD > 75%) (Figure S2). IBD partitioned by geographical location showed 44.8% sample pairs of Loreto–Loreto, 44% of Loreto–Tumbes and 11.2% of Tumbes–Tumbes. In terms of time, there were 13.8% of IBD sample pairs from 2006/2010, 32.8% of IBD sample pairs from 2006/2010–2011/2017 and 53.4% of IBD sample pairs from 2011/2017 (Figure S2). In terms of drug resistance, 95.6% of IBD sample pairs were among Bv1 type samples.

The IBD network showed clustering according to the typed lineages (Bv1, Acre-Loreto and Clonet D) as well as the link between Loreto and Tumbes samples (Figure S3). Furthermore, IBD confirmed the population structure results from PCA and admixture and the hypothesis of Bv1 expansion as all sample pairs with more than 25% of the genome on IBD collected after 2011 in Loreto were among Bv1 (Figures S3and S4).

### Molecular analyses of PfS47 gene

**Figure 6:**
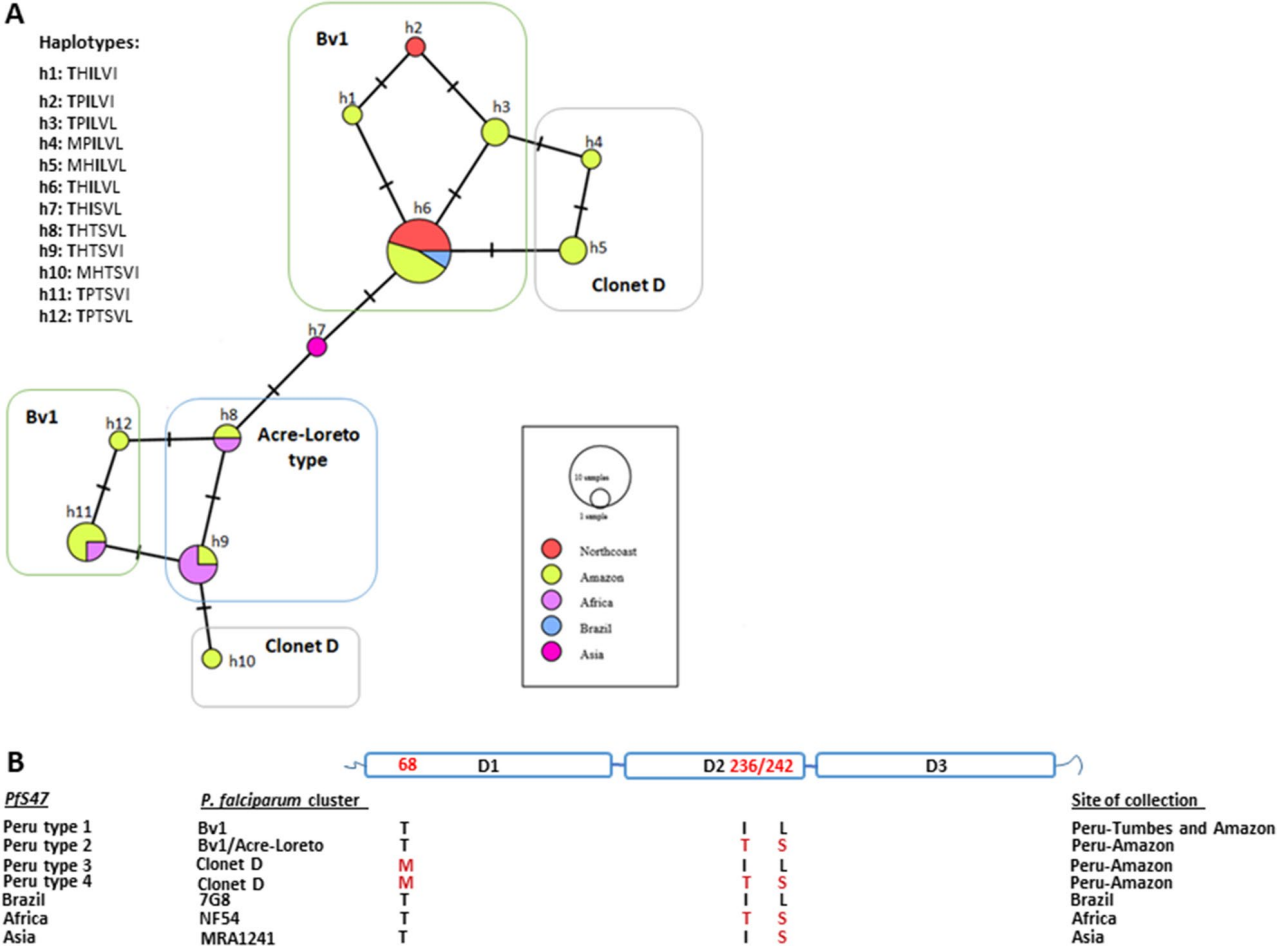
Structural diversity of *pfS47* (**A**) Haplotype network for Peruvian isolates and control samples from Brazil, Africa and Asia. The network shows the distribution of the typed Peruvian lineages according to their *pfS47* haplotype. (**B**) Four *PfS47* circulating haplotypes in Peru based on three amino acid positions associated with evasion to mosquito immune response. D1, D2 and D3 represents protein domains and 68, 236 and 242 amino acid positions.

Peruvian samples were grouped into 11 *PfS47* haplotypes of nonsynonymous mutations (Fig. 6, Table S5). The most prevalent haplotype was THILVL at positions 68, 194, 236, 242, 247 and 248 that was prevalent in 42% of the Peruvian samples (n = 10) (Fig. 6A). Control samples from Africa and Brazil were grouped into four haplotypes together with some of the Peruvian samples whereas a control reference from Asia (MRA1241) separated into a unique haplotype.

We found amino acid changes in two (236, 242) of the three (236, 242, 247) positions associated with invasion of mosquitos reported in previous studies^49^. We also found a change in position 68 with the amino acid threonine in all samples collected in Tumbes whereas samples from the Peruvian Amazon Basin presented threonine or methionine in this position (Fig. 6B).

Using the amino acid changes on regions associated with mosquito response evasion, we identified four *PfS47* haplotypes in Peru named type 1 (TIL), type 2 (TTS), type 3 (MIL) and type 4 (MTS). The type 1 haplotype was found in seven Bv1-type samples isolated from the Peruvian Amazon Basin and the North Coast. The type 2 haplotype was found on four Bv1-types and one Acre-Loreto isolates from the Peruvian Amazon Basin. The type 3 and 4 corresponded to one Cloned D-type each isolated from the Peruvian Amazon Basin (Figure S5). The *PfS47* Brazilian control presented the same structure as Peru type 1 whereas the Africa control the same as Peru type 2 (Fig. 6B). Analysis of diversity and selection showed that *PfS47* in Peru is under positive selection (Tajima´s D = 1.44) although with a low diversity (π = 0.0015).

## Discussion

Evidence of positive selection on surface genes mediating host cell surface binding are an important result from this study and supports previous studies that indicate that immune pressure on parasites leads to positive selection on diversity maintaining the pool of heterozygotes in the population^50^. This adaptive mechanism could have important consequences for the development of effective vaccines against these parasites due to the risk of high polymorphism on putative vaccine candidates.

However, it is important to note that Peru is a low malaria transmission region with low parasite recombination rates and highly clonal populations^15,17,51^. These populations are prone to demographic size changes derived from introduction, drug selection pressure, malaria control, eradication strategies and genetic drift as previously shown^15,18^. Therefore, results derived from neutral hypothesis tests such as Tajima´s D test should be interpreted with caution^52^.

During the last three decades, malaria rates in Peru have fluctuated overtime because of malaria control initiatives, changes in treatment regimens, migration and climatic variables. These different factors have exerted selection pressure on the parasite population. Our results of molecular drug resistance profiles show that although SP and CQ are no longer used to treat *P. falciparum* in Peru, drug resistance associated mutations in *pfmdr1* and *pfcrt* appear to be maintained in the parasite population pool throughout time. In this regard, it has been previously suggested that the low prevalence of *pfcrt* wild type strains would limit the recovery of sensitive CQ resistant parasites even in the absence of CQ^15,53^ in contrast to what has been shown in Africa^54^.

However, it is also possible that *P. falciparum* strains in Peru are subjected to indirect selection pressures to CQ as a result of CQ based treatment for *P. vivax* infections. Therefore, *P. falciparum* could be exposed to CQ during treatment of misdiagnosed mixed infections or as a result of inadequate diagnosis. In the case of SP, the majority of the Bv1-type samples were triple mutants *for pfdhfr* and *pfdhps* which is consistent with the first reports of *P. falciparum* foreign lineages in the Peruvian territory^55^.

None of the tested samples presented mutations associated to artemisinin resistance in *pfk13*. This result is consistent with reports from other countries in the region and is an important finding given the threat of potential emergence of artemisinin resistance in the Guyana shield^56^ and the high migration rates across all the region as a result of the Venezuela crisis and illegal activities conducted across the triple border of Brazil, Venezuela and Guayana^57,58^. Circulating Peruvian strains harbor low frequency polymorphisms outside the propeller domain such as the K189T mutation that has been originally reported in Africa and subsequently in Brazil, Venezuela and French Guyana^59,60^.

Our study has shown the presence to three *P. falciparum* subpopulations one of which (Bv1-type) was present in both regions whereas the other two were present only in the Peruvian Amazon. These lineages seem to be related to previously reported clonets that appeared to be widespread in Peru^15,55^. The long-term persistence of these lineages for near two decades could be linked to a bottleneck that resulted from the strong selective pressure from the introduction of artesunate combination therapies in Peru, the low mosquito Human Blood Index and the characteristics of the Peru as a low transmission region in contrast with transmission patterns seen in Africa. This finding is further supported by the low number of polyclonal infections, which reduce the chances of genetic outcrossing and recombination. Therefore, these characteristics of malaria transmission in Peru limit increased parasite diversity or the emergence of more virulent and resistant strains.

The Bv1-*dhfr* profile (50R/51I/108N) was found by the first time in isolates from Bolivia (1994) and then in samples from Brazil (1997) and Venezuela (1998)^61^. Studies in Peru did not report the Bv1-*dhfr* profile until 2006 when it was detected in samples from the Amazon Basin^55^. Our population structure and phylogenetic analysis show that this Bv1-type lineage has become predominant in the Peruvian Amazon Basin since 2011.This lineage presents several drug resistance associated polymorphisms, which might have provided a selective advantage to the indirect selective pressure of CQ for treatment for *P. vivax* in the region. In this regard, further studies with increased sample size and geographic coverage are needed to confirm the Bv1-type lineage expansion in the region.

The Bv1 line has a history of contributing to two major outbreaks in Peru. The first one in in the Peruvian North Coast in 2010–2012^17^ and the other one in the region of Cusco in 2013^18^. Interestingly, the potential strain replacement suggested by our study in the Peruvian Amazon occurred during a period of rapid increase in the number of *P. falciparum* cases in the Amazon Basin raising from 2600 cases to 10,200 between 2011 and 2014.

The potential spread and increase in the number of cases could be due to the fact that the Bv1 line presents a high frequency of *pfhrp2* and *pfhrp3* gene deletions^17,18^. Our data further indicates that deletions have been sustained overtime with none of the more recent Bv1 samples presenting coverage for both genes. The absence of *pfhrp2* and *pfhrp3* constitutes a major challenge for malaria elimination in Peru given the use of RDTs for malaria diagnosis. In this regard, previous studies conducted in Peru have shown up to 41% of *pfhrp2* gene deletions and RDTs sensitivities between 50 to 70% compared to microscopy or PCR^62,63^.

The potential replacement of circulating lineages by the Bv1 lineage parasites in Loreto and Colombia where it has been previously reported^64,65^ seems to suggest that this line is highly adaptable to different transmission settings. In this regard, it is important to point out that *Anopheles darlingi* and *Anopheles oswaldoi* are reported malaria vectors in the Amazonas Department in Colombia^66^ whereas *Anopheles darlingi* and *Anopheles benarrochi* are the among the main vectors in the Peruvian Amazon Basin. In contrast, *Anopheles albimanus* is the main vector in the Peruvian North coast and *Anopheles pseudopunctipennis* in the Department of Cusco. Therefore, there is evidence to point out that the Bv1-type lineage is able to adapt to a wide range of malaria vectors that could allow it to expand in different endemic settings.

The mosquito immune system have an important role preventing the transmission of the parasite. In response to this threat, the parasite evades mosquito immune response through different mechanisms. One of these mechanisms appears to be linked to the *PfS47* gene that allows the parasite to evade mosquito immune detection by disrupting JNK signaling in invaded midgut cells and blocking TEP1-mediated killing^67,68^.

In vivo studies associated that some *PfS47* haplotypes can mediate the invasion of different anopheline species evading their immune response^69,70^. Furthermore, genetic studies have shown evidence of *PfS47* mediated vector-parasite compatibility that limits the parasite to specific vector species^70^ in a “lock-and-key” model. This limitation is in an important barrier for *P. falciparum* infection and transmission as a result of purifying selection mediated by anopheline species^70^.

In this regard, we report three positions on *PfS47* gene (68, 236 and 242) that are probably associated with evasion of the anopheline´s immune response in our region. Out of the four Peruvian *PfS47* haplotypes the *Pfs47* type1 (TIL) was detected in the North Coast and the Amazon Basin. Furthermore, the Brazilian control *PfS47* haplotype was similar to the Peruvian *PfS47* type1 whereas the African control the same as the Peruvian *PfS47* type 2. The compatibility of *PfS47* haplotypes with the Brazilian lineage is in alignment with hypothesis of introduction of Bv1 from Brazil around 2006 (V. Udhayakumar, unpublished) and highlights the possibility of future introduction of drug resistant lineages from Brazil or Africa.

The presence of Bv1 in areas with different malaria vectors highlight the need to conduct further research to explore the *PfS47* gene variants of and other virulence markers that could have facilitated the spread of Bv1 through Peru and other endemic regions.

Although our sample size is rather limited, our findings provide grounds for additional studies with larger sample sizes that could provide additional evidence of this change in the parasite population in Peru. In this regard, further research is needed to assess the effects of recent migration waves, malaria control activities and the impact of COVID19 on public health in the transmission of malaria in Peru.

## Disclaimer

The views expressed in this article are those of the authors and do not necessarily reflect the official policy or position of the Department of the Navy, Department of Defense, nor the U.S. Government.

## Copyright

Several authors of this paper are employees of the U.S. Government. This work was prepared as part of their official duties. Title 17 U.S.C. §105 provides that ‘Copyright protection under this title is not available for any work of the United States Government.’ Title 17 U.S.C. §101 defines a U.S. Government work as a work prepared by a military service member or employee of the U.S. Government as part of that person’s official duties.

## Supplementary Information

Supplementary Information.

## Data Availability

The dataset generated during and/or analyzed during the current study is available from the corresponding author on reasonable request. Raw sequence data has been deposited at the European Nucleotide Archive (https://www.ebi.ac.uk/ena/browser/home) under primary accession number PRJEB46168.
