# Supplementary material for: Population genomics and evidence of clonal replacement of Plasmodium falciparum in the Peruvian Amazon
Source: Sci Rep. 2021 Oct 27;11:21212. doi: 10.1038/s41598-021-00806-5 (PMC8551272; doi:10.1038/s41598-021-00806-5)
Supplement: Supplementary file 1 — Supplementary Information. [file 41598_2021_806_MOESM1_ESM.pdf]

Supplementary files

Population genomics and evidence of clonal replacement of *Plasmodium falciparum* in the Peruvian Amazon

Fredy E. Villena, Stephen E. Lizewski, Christie A. Joya, Hugo O. Valdivia

S1 Table. Next generation sequencing metrics. The table shows coverage and read depth metrics for each of the samples.

Sample	Year of collection	Region	Mean Coverage	Median Coverage	% Core genome covered by >=5 reads	*P. falciparum* reads	Human Reads	%*P. falciparum* reads
CDR20008	2010	North Coast	17.67	8	61.83	2331975	36705	98.45
CDR20009	2010	North Coast	16.90	4	45.96	1774293	195107	90.09
CDR20001	2010	North Coast	22.09	7	58.56	2725774	89890	96.81
CDR20003	2010	North Coast	16.04	0	24.27	2011536	534096	79.02
CDR20002	2011	North Coast	25.49	8	59.28	3763286	95890	97.52
CDR20005	2011	North Coast	34.68	8	58.45	4595900	209395	95.64
CDR20010	2006	Northern Amazon Basin	25.04	13	70.48	2637423	29751	98.88
CDR20011	2007	Northern Amazon Basin	21.07	5	52.87	2292297	242698	90.43
MDP2017	2009	Northern Amazon Basin	24.02	10	64.88	2431705	93439	96.30
MDP2018	2009	Northern Amazon Basin	28.03	15	71.84	2871501	34272	98.82
CDR20012	2010	Northern Amazon Basin	20.01	4	46.51	2988724	437382	87.23
CDR20013	2010	Northern Amazon Basin	26.69	11	66.55	2930233	285110	91.13
MDP3015	2010	Northern Amazon Basin	25.52	9	64.05	2734368	16203	99.41
MDP3120	2011	Northern Amazon Basin	29.84	5	50.53	3281114	190920	94.50
MDP3130	2011	Northern Amazon Basin	22.87	6	53.96	2780773	134997	95.37
MDP3207	2012	Northern Amazon Basin	28.78	11	66.56	3053698	7626	99.75
MDP5048	2013	Northern Amazon Basin	37.01	16	73.09	4135451	190890	95.59
CDR20014	2014	Northern Amazon Basin	20.64	11	67.68	2233106	47704	97.91
MDP3823	2014	Northern Amazon Basin	7.77	2	31.13	843522	79052	91.43
MDP3860	2015	Northern Amazon Basin	7.18	2	38.20	724615	21604	97.10
MDP3895	2015	Northern Amazon Basin	7.92	0	19.01	865262	29440	96.71
MDP3962	2016	Northern Amazon Basin	7.77	2	31.13	774647	68704	91.85
MDP3988	2016	Northern Amazon Basin	9.01	3	39.48	901626	43537	95.39
MDP6123	2017	Northern Amazon Basin	9.26	3	42.12	978523	40711	96.01

S2 Table. Gene Ontology analysis for high SNP density regions on chromosomes 1, 2, 4, 5, 7, 8, 11, 13 and 14. P-value after Bonferroni correction.

GO-Id	Methodology	p-value	GO Description	Gene Id
46812	Tajima´s D	2.55E-02	Host cell surface binding	PF3D7_0100400; PF3D7_0113800; PF3D7_0533100; PF3D7_0808700; PF3D7_1200600; PF3D7_1371600
5516	SnpEff	3.73E-03	Calmodulin binding	PF3D7_0506500; PF3D7_0529800; PF3D7_1239800; PF3D7_1320700; PF3D7_1325400; PF3D7_1444100; PF3D7_1464500
3779	SnpEff	8.26E-03	Actin binding	PF3D7_0424400; PF3D7_0506500; PF3D7_0529800; PF3D7_1239800; PF3D7_1320700; PF3D7_1325400; PF3D7_1444100 PF3D7_1464500
46812	SnpEff	8.67E-03	Host cell surface binding	PF3D7_0102500; PF3D7_0113800; PF3D7_0402300; PF3D7_0424400; PF3D7_0533100; PF3D7_0731500; PF3D7_0930300; PF3D7_1133400; PF3D7_1149000; PF3D7_1252100; PF3D7_1335100; PF3D7_1335900
1901681	SnpEff	3.84E-02	Sulfur compound binding	PF3D7_0102500; PF3D7_0315200; PF3D7_0402300; PF3D7_0731500; PF3D7_1469600
3774	SnpEff	4.49E-02	Motor activity	PF3D7_0506500; PF3D7_0529800; PF3D7_1025500; PF3D7_1239800; PF3D7_1320700; PF3D7_1325400; PF3D7_1444100; PF3D7_1464500

S3 Table. Tajima's D sliding window result. The table shows genes located in regions under positive or negative selection.

Chromosome	Window location	Tajima's D	Gene IDs	Gene (window 5000bp)
1	535000	2.01172	PF3D7_0113800; PF3D7_0113900	DBL containing protein, unknown function
1	70000	2.06658	PF3D7_0101100; PF3D7_0101200; PF3D7_0101300	exported protein family 4 / exported protein family 3 / Pfmc-2TM Maurer's cleft two transmembrane protein
1	50000	2.9162	PF3D7_0100400; PF3D7_0100500; PF3D7_0100600	Rifin / erythrocyte membrane protein 1 (PfEMP1), exon 1, pseudogene
2	60000	2.26224	PF3D7_0201100; PF3D7_0201200	Plasmodium exported protein, unknown function, fragment / rifin
2	40000	2.29309	PF3D7_0200500	rifin
2	105000	2.43758	PF3D7_0202000; PF3D7_0202100	knob-associated histidine-rich protein / liver stage associated protein 2
2	50000	2.62814	PF3D7_0200700; PF3D7_0200800; PF3D7_0200900	Rifin / erythrocyte membrane protein 1 (PfEMP1), exon 2, pseudogene / stevor, pseudogene
4	1145000	2.08736	PF3D7_0425500; PF3D7_0425600	Stevor / rifin, pseudogene
5	1295000	2.06658	PF3D7_0532000	28S ribosomal RNA
5	1155000	2.22585	PF3D7_0527900; PF3D7_0528000; PF3D7_0528100	ATP-dependent RNA helicase DDX41, putative / proteasome maturation factor UMP1, putative / AP-1 complex subunit beta, putative
5	1320000	2.27401	PF3D7_0532700; PF3D7_0532800	erythrocyte membrane protein 1 (PfEMP1), pseudogene / stevor, pseudogene
5	1335000	2.39943	PF3D7_0533100	erythrocyte membrane protein 1 (PfEMP1), pseudogene
5	1340000	3.35781	PF3D7_0533100	erythrocyte membrane protein 1 (PfEMP1), pseudogene
6	1335000	2.34562	PF3D7_0632000; PF3D7_0632100	Rifin / rifin
8	1430000	2.06658	PF3D7_0833300; PF3D7_0833400	erythrocyte membrane protein 1 (PfEMP1), exon 2, pseudogene / rifin
8	445000	2.29309	PF3D7_0808700	erythrocyte membrane protein 1, PfEMP1
8	1310000	2.30835	PF3D7_0830800	surface-associated interspersed protein 8.2 (SURFIN 8.2)
8	1305000	2.4399	PF3D7_0830700	Plasmodium exported protein (hyp9), unknown function
8	1370000	2.48411	PF3D7_0831750; PF3D7_0831800	Plasmodium exported protein (PHISTa), unknown function, pseudogene / histidine-rich protein II
8	70000	2.65574	PF3D7_0800700	surface-associated interspersed protein 8.3 (SURFIN 8.3)
10	1510000	2.12625	PF3D7_1038100; PF3D7_1038200; PF3D7_1038300	GDP dissociation inhibitor, putative / conserved Plasmodium protein, unknown function / conserved Plasmodium protein, unknown function
10	1515000	2.14883	PF3D7_1038400	gametocyte-specific protein
10	50000	2.26224	PF3D7_1000600; PF3D7_1000700	Rifin / Plasmodium exported protein (PHISTa), unknown function, pseudogene
10	55000	2.47823	PF3D7_1000700; PF3D7_1000800	Plasmodium exported protein (PHISTa), unknown function, pseudogene / stevor, pseudogene
10	1390000	2.52818	PF3D7_1035100; PF3D7_1035200	probable protein, unknown function / S-antigen
10	1395000	3.17436	PF3D7_1035200; PF3D7_1035300	S-antigen / glutamate-rich protein GLURP
11	1940000	2.78334	PF3D7_1148800	Plasmodium exported protein (hyp11), unknown function
12	465000	2.01676	PF3D7_1210300; PF3D7_1210400	conserved Plasmodium protein, unknown function / general transcription factor 3C polypeptide 5, putative
12	2215000	2.29309	PF3D7_1254400; PF3D7_1254500	Rifin / rifin
12	2190000	2.53794	PF3D7_1253700	rifin
12	55000	2.74669	PF3D7_1200600	erythrocyte membrane protein 1, PfEMP1
13	2820000	2.21155	PF3D7_1371600; PF3D7_1371700; PF3D7_1371800	erythrocyte binding like protein 1, pseudogene / serine/threonine protein kinase, FIKK family / Plasmodium exported protein, unknown function
13	2840000	2.29309	PF3D7_1372200	histidine-rich protein III
13	65000	2.29309	PF3D7_1301000; PF3D7_1301100	rifin, pseudogene / Plasmodium exported protein (PHISTa), unknown function, pseudogene
13	2485000	2.41942	PF3D7_1362000	cytochrome c oxidase subunit ApiCOX24, putative / 14-3-3 protein
14	3285000	2.06658	PF3D7_1480000	rifin
14	10000	2.73352	PF3D7_1400300; PF3D7_1400400	Rifin / rifin
3	885000	-2.10801	PF3D7_0321100; PF3D7_0321200	conserved Plasmodium protein, unknown function / UDP-N-acetylglucosamine--dolichyl-phosphate N-acetylglucosaminephosphotransferase, putative
3	575000	-2.18868	PF3D7_0314200; PF3D7_0314300; PF3D7_0314400	conserved Plasmodium protein, unknown function
4	735000	-2.02076	PF3D7_0416900	conserved Plasmodium protein, unknown function
6	580000	-2.06261	PF3D7_0613900	myosin E, putative
6	195000	-2.16902	PF3D7_0604500; PF3D7_0604600	conserved Plasmodium protein, unknown function / DNA helicase, putative
6	610000	-2.25201	PF3D7_0614600; PF3D7_0614700	conserved Plasmodium protein, unknown function
6	805000	-2.35793	PF3D7_0619300	conserved Plasmodium protein, unknown function
8	935000	-2.06104	PF3D7_0820700; PF3D7_0820800; PF3D7_0820900	2-oxoglutarate dehydrogenase E1 component / conserved Plasmodium protein, unknown function
8	1165000	-2.16401	PF3D7_0826900; PF3D7_0827000	conserved Plasmodium protein, unknown function / ATP-dependent RNA helicase DBP10, putative
9	530000	-2.04326	PF3D7_0911600; PF3D7_0911700	conserved Plasmodium protein, unknown function / GTP-binding protein, putative
9	1060000	-2.09715	PF3D7_0926200; PF3D7_0926300	conserved Plasmodium protein, unknown function / protein kinase, putative
9	1110000	-2.14292	PF3D7_0927200; PF3D7_0927300	zinc finger protein, putative / fumarate hydratase
9	920000	-2.14756	PF3D7_0922700; PF3D7_0922800	pre-mRNA-splicing factor 18, putative / conserved Plasmodium protein, unknown function
10	880000	-2.36214	PF3D7_1021700	conserved Plasmodium membrane protein, unknown function
11	430000	-2.09148	PF3D7_1110700; PF3D7_1110800; PF3D7_1110900; PF3D7_1111000	actin-like protein, putative / conserved Plasmodium protein, unknown function / ES2 protein, putative / tRNA m5C-methyltransferase, putative
11	425000	-2.10032	PF3D7_1110500; PF3D7_1110600; PF3D7_1110700	vacuolar protein sorting-associated protein 35, putative / mitochondrial ribosomal protein L11 precursor, putative / actin-like protein, putative
12	1505000	-2.03291	PF3D7_1236100	clustered-asparagine-rich protein
12	890000	-2.18349	PF3D7_1222400	AP2 domain transcription factor
13	1930000	-2.08837	PF3D7_1348300	elongation factor Tu, putative
13	1900000	-2.12222	PF3D7_1347600	conserved protein, unknown function
13	1790000	-2.21894	PF3D7_1344600; PF3D7_1344700	lipoyl synthase / conserved Plasmodium protein, unknown function
14	1310000	-2.06074	PF3D7_1433300; PF3D7_1433400	chromatin assembly factor 1 P55 subunit, putative / zinc finger protein, putative
14	520000	-2.23828	PF3D7_1412900; PF3D7_1413000	ubiquitin-conjugating enzyme E2, putative / ubiquitin-conjugating enzyme E2, putative / conserved Plasmodium protein, unknown function
14	1110000	-2.24373	PF3D7_1428400	WD and tetratricopeptide repeats protein 1, putative

S4 Table. Table of variants impact that shows the effect produced by nucleotide changes on genetic regions as determined by SnpEff. High: The variant is assumed to have high (disruptive) impact in the protein. This probably results in protein truncation, loss of function or triggering nonsense mediated decay. Moderate: A non-disruptive variant that might change protein effectiveness.

Family	Gene	Gene ID	Varian Impact
-	ring-exported protein 2	PF3D7_0936000	High
AMP-dependent synthetase/ligase	acyl-CoA synthetase	PF3D7_0301000	High
Protein kinase-like domain superfamily	conserved Plasmodium protein, unknown function	PF3D7_1303800	High
-	surface-associated interspersed protein 1.2 (SURFIN 1.2), pseudogene	PF3D7_0113600	High
-	surface-associated interspersed protein 4.1 (SURFIN 4.1), pseudogene	PF3D7_0402200	High
-	Metacaspase-2	PF3D7_1438400	High
-	conserved Plasmodium protein, unknown function	PF3D7_1320700	High
-	gamete egress and sporozoite traversal protein, putative	PF3D7_1449000	High
Sec7 domain	protein transport protein SEC7, putative	PF3D7_1442900	High
Variant surface antigen Rifin	rifin	PF3D7_0114700	High
Inositol polyphosphate kinase	inositol polyphosphate multikinase, putative	PF3D7_0514800	High
-	conserved Plasmodium protein, unknown function	PF3D7_0806100	High
Peptidase C2, calpain, large subunit, domain III	calpain	PF3D7_1362400	High
Plasmodium RESA, N-terminal	Plasmodium exported protein (PHISTb), unknown function	PF3D7_1476300	High
Schizont-infected cell agglutination, C-terminal domain	surface-associated interspersed protein 8.1 (SURFIN 8.1)	PF3D7_0831100	High
DEAD/DEAH box helicase domain	Stevor-like	PF3D7_0310500	High
Helicase superfamily 1/2, ATP-binding domain	ATP-dependent RNA helicase DBP10, putative	PF3D7_0827000	High
-	Plasmodium exported protein, unknown function	PF3D7_0221700	High
-	conserved Plasmodium protein, unknown function	PF3D7_0933100	High
-	conserved Plasmodium protein, unknown function	PF3D7_0828100	High
-	conserved Plasmodium protein, unknown function	PF3D7_1018900	High
Exported protein, PHISTa/b/c, conserved domain, Plasmodium	Plasmodium exported protein (PHISTa-like), unknown function	PF3D7_0832200	High
Predicted 26S proteasome regulatory complex, non-ATPase subcomplex, subunit s5a, Plasmodium	26S proteasome regulatory subunit RPN10, putative	PF3D7_0807800	High
-	Plasmodium exported protein, unknown function	PF3D7_0220300	High
Variant surface antigen Stevor	Stevor-like	PF3D7_0300900	High
Serine-threonine/tyrosine-protein kinase, catalytic domain	serine/threonine protein kinase, FIKK family	PF3D7_0902100	High
Acetyl-CoA carboxylase	metalloprotease, putative	PF3D7_1009500	High
-	conserved Plasmodium protein, unknown function	PF3D7_0620200	High
-	conserved Plasmodium membrane protein, unknown function	PF3D7_0625500	High
Zinc finger, CCCH-type	zinc finger protein, putative	PF3D7_1425600	High

-	Plasmodium exported protein, unknown function	PF3D7_0221000	High
-	conserved Plasmodium protein, unknown function	PF3D7_0323800	High
-	conserved Plasmodium protein, unknown function	PF3D7_1303800	Moderate
-	conserved Plasmodium protein, unknown function	PF3D7_0417400	Moderate
Apical membrane antigen 1	apical membrane antigen 1	PF3D7_1133400	Moderate
-	KELT protein	PF3D7_1475900	Moderate
-	DBL containing protein, unknown function	PF3D7_0113800	Moderate
Schizont-infected cell agglutination, C-terminal domain	surface-associated interspersed protein 8.2 (SURFIN 8.2)	PF3D7_0830800	Moderate
Merozoite surface 1, C-terminal	merozoite surface protein 1	PF3D7_0930300	Moderate
-	conserved Plasmodium membrane protein, unknown function	PF3D7_0703900	Moderate
-	conserved Plasmodium protein, unknown function	PF3D7_0418000	Moderate
Zinc finger, CW-type	zinc finger protein, putative	PF3D7_0420000	Moderate
Mediator complex, subunit Med14	Cg2 protein	PF3D7_0709300	Moderate
-	conserved Plasmodium membrane protein, unknown function	PF3D7_0804500	Moderate
-	conserved Plasmodium protein, unknown function	PF3D7_1475800	Moderate
-	probable protein, unknown function	PF3D7_1035100	Moderate
6-Cysteine (6-Cys) domain	6-cysteine protein P230	PF3D7_0209000	Moderate
Protein kinase-like domain superfamily	phosphatidylinositol 4-kinase, putative	PF3D7_0419900	Moderate
DNA (cytosine-5)-methyltransferase 1, replication foci domain	conserved Plasmodium protein, unknown function	PF3D7_1448500	Moderate
Thrombospondin type-1 (TSP1) repeat	thrombospondin-related anonymous protein	PF3D7_1335900	Moderate
-		PF3D7_0402200	Moderate
Peptidase C50, separase	peptidase family C50, putative	PF3D7_0809600	Moderate
Cytosine specific DNA methyltransferase replication foci domain	conserved protein, unknown function	PF3D7_1408700	Moderate
-	conserved Plasmodium protein, unknown function	PF3D7_0710200	Moderate
-	reticulocyte binding protein homologue 1	PF3D7_0402300	Moderate
-	surface-associated interspersed protein 4.2 (SURFIN 4.2)	PF3D7_0424400	Moderate
-	conserved Plasmodium protein, unknown function	PF3D7_0710000	Moderate
-	conserved Plasmodium protein, unknown function	PF3D7_0713900	Moderate
-	conserved Plasmodium membrane protein, unknown function	PF3D7_0212400	Moderate
-	Plasmodium exported protein, unknown function	PF3D7_1201400	Moderate
-		PF3D7_1438400	Moderate
-	HECT-domain (ubiquitin-transferase), putative	PF3D7_0628100	Moderate
Papain-like cysteine peptidase superfamily	ubiquitin carboxyl-terminal hydrolase 1, putative	PF3D7_0104300	Moderate

-	conserved Plasmodium protein, unknown function	PF3D7_1308400	Moderate
-	protein SOC2, putative	PF3D7_1227500	Moderate
-	erythrocyte membrane-associated antigen	PF3D7_0422200	Moderate
Acetyl-CoA carboxylase	acetyl-CoA carboxylase	PF3D7_1469600	Moderate
Trimeric LpxA-like superfamily	conserved Plasmodium protein, unknown function	PF3D7_0504800	Moderate
-	conserved Plasmodium protein, unknown function	PF3D7_0522400	Moderate
Duffy-antigen binding	erythrocyte binding antigen-181	PF3D7_0102500	Moderate
-	conserved Plasmodium protein, unknown function	PF3D7_1239800	Moderate
SET domain	SET domain protein, putative	PF3D7_0629700	Moderate
-	conserved Plasmodium protein, unknown function	PF3D7_1325400	Moderate
-	Plasmodium exported protein, unknown function	PF3D7_0701900	Moderate
Hemimethylated DNA-binding domain superfamily	Cg1 protein	PF3D7_0709100	Moderate
Duffy binding domain	regulator of chromosome condensation-PP1-interacting protein	PF3D7_0919900	Moderate
Vacuolar protein sorting-associated protein 13, N-terminal domain	conserved Plasmodium membrane protein, unknown function	PF3D7_1021700	Moderate
HECT, E3 ligase catalytic domain	E3 ubiquitin-protein ligase	PF3D7_0704600	Moderate
Cytoadherence-linked asexual protein	cytoadherence linked asexual protein 2	PF3D7_0220800	Moderate
-	HECT-like E3 ubiquitin ligase, putative	PF3D7_0826100	Moderate
-	conserved Plasmodium membrane protein, unknown function	PF3D7_0726400	Moderate
-	conserved Plasmodium protein, unknown function	PF3D7_1444100	Moderate
Regulator of chromosome condensation (RCC1) repeat	cysteine repeat modular protein 4	PF3D7_1475400	Moderate
HECT-domain (ubiquitin-transferase)	cation-transporting ATPase 1	PF3D7_0516100	Moderate
Cytoadherence-linked asexual protein	cysteine repeat modular protein 1	PF3D7_0911300	Moderate
HECT-domain (ubiquitin-transferase)	conserved Plasmodium protein, unknown function	PF3D7_1237100	Moderate
-	conserved Plasmodium membrane protein, unknown function	PF3D7_1474200	Moderate
Thrombospondin type-1 (TSP1) repeat	circumsporozoite- and TRAP-related protein	PF3D7_0315200	Moderate
-	conserved Plasmodium membrane protein, unknown function	PF3D7_0806700	Moderate
GroEL-like apical domain superfamily	conserved Plasmodium protein, unknown function	PF3D7_1412400	Moderate
P5-type ATPase cation transporter	leucine-rich repeat protein	PF3D7_0612200	Moderate
Kringle	conserved Plasmodium protein, unknown function	PF3D7_0916400	Moderate
6-Cysteine (6-Cys) domain	liver specific protein 2, putative	PF3D7_0405300	Moderate
Merozoite surface protein, C-terminal	merozoite surface protein 7	PF3D7_1335100	Moderate
Thrombospondin type-1 (TSP1) repeat	Plasmodium exported protein, unknown function	PF3D7_1352900	Moderate
Pseudouridine synthase	RNA pseudouridylate synthase, putative	PF3D7_0511500	Moderate

GroEL-like apical domain superfamily	erythrocyte membrane protein 1 (PfEMP1), pseudogene	PF3D7_0533100	Moderate
Leucine Rich repeats	conserved Plasmodium protein, unknown function	PF3D7_1025500	Moderate
AP2/ERF domain	AP2 domain transcription factor, putative	PF3D7_0613800	Moderate
-	sporozoite and liver stage asparagine-rich protein	PF3D7_1147000	Moderate
Merozoite surface protein, C-terminal	tryptophan-rich antigen 3	PF3D7_1002200	Moderate
Helicase/SANT-associated domain	conserved Plasmodium protein, unknown function	PF3D7_1417600	Moderate
MATH/TRAF domain	surface-associated interspersed protein 1.1 (SURFIN 1.1)	PF3D7_0113100	Moderate
-	conserved Plasmodium protein, unknown function	PF3D7_0307900	Moderate
Dynein heavy chain, domain 2, C-terminal	conserved Plasmodium protein, unknown function	PF3D7_1421100	Moderate
AP2/ERF_dom	conserved Plasmodium protein, unknown function	PF3D7_0531100	Moderate
-	conserved Plasmodium membrane protein, unknown function	PF3D7_0704000	Moderate
Tryptophan/threonine-rich plasmodium antigen, C-terminal	zinc finger protein, putative	PF3D7_0615600	Moderate
Helicase/SANT-associated domain	erythrocyte binding antigen-175	PF3D7_0731500	Moderate
Schizont-infected cell agglutination, C-terminal domain	AP2 domain transcription factor AP2-G	PF3D7_1222600	Moderate
Haem oxygenase-like, multi-helical	conserved Plasmodium protein, unknown function	PF3D7_0526600	Moderate
Rab-GTPase-TBC domain superfamily	eukaryotic translation initiation factor 2-alpha kinase	PF3D7_0628200	Moderate
Zinc finger, RING/FYVE/PHD-type	conserved Plasmodium protein, unknown function	PF3D7_0713500	Moderate
Summary: Erythrocyte binding antigen 175	conserved Plasmodium protein, unknown function	PF3D7_1343800	Moderate
AP2/ERF domain	nucleoporin NUP637, putative	PF3D7_0609000	Moderate
ARID DNA-binding domain	conserved Plasmodium protein, unknown function	PF3D7_0603600	Moderate
Trimeric LpxA-like superfamily	rhoptry neck protein 3	PF3D7_1252100	Moderate
Protein kinase domain	conserved Plasmodium protein, unknown function	PF3D7_0529800	Moderate
Zinc finger, PHD-type, conserved site	zinc finger protein, putative	PF3D7_1008100	Moderate
Zinc finger, CCCH-type	conserved Plasmodium protein, unknown function	PF3D7_1207200	Moderate
MORN motif	MORN repeat protein, putative	PF3D7_1306500	Moderate
Vacuolar protein sorting-associated protein 13	dynein-related AAA-type ATPase, putative	PF3D7_1434500	Moderate
RP1/RP1L1/DCX	asparagine-rich antigen Pfa35-2	PF3D7_0105700	Moderate
ARID DNA-binding domain	patatin-like phospholipase, putative	PF3D7_0218600	Moderate
-	oocyst capsule protein Cap380	PF3D7_0320400	Moderate
-	conserved Plasmodium protein, unknown function	PF3D7_0506500	Moderate
Zinc finger, PHD-type	conserved Plasmodium protein, unknown function	PF3D7_0703200	Moderate
ATPase, dynein-related, AAA domain	acyl-CoA synthetase	PF3D7_0215300	Moderate
-	conserved Plasmodium protein, unknown function	PF3D7_1136000	Moderate
-		PF3D7_1320700	Moderate
-	conserved Plasmodium protein, unknown function	PF3D7_0421700	Moderate
-	conserved Plasmodium protein, unknown function	PF3D7_1140900	Moderate

Cleavage/polyadenylation specificity factor, A subunit, C-terminal	conserved Plasmodium protein, unknown function	PF3D7_0513200	Moderate
-	conserved Plasmodium protein, unknown function	PF3D7_0711200	Moderate
-	conserved Plasmodium protein, unknown function	PF3D7_0317300	Moderate
K Homology domain, type 1 superfamily	conserved protein, unknown function	PF3D7_0510100	Moderate
AMP-binding enzyme	histone-lysine N-methyltransferase SET2	PF3D7_1322100	Moderate
NOL6/Upt22	conserved Plasmodium protein, unknown function	PF3D7_1422400	Moderate
WD40-repeat-containing domain superfamily	conserved Plasmodium membrane protein, unknown function	PF3D7_0619800	Moderate
-	conserved Plasmodium protein, unknown function	PF3D7_1457400	Moderate
-	conserved Plasmodium protein, unknown function	PF3D7_1019100	Moderate
-	conserved Plasmodium protein, unknown function	PF3D7_1474000	Moderate
-	conserved Plasmodium membrane protein, unknown function	PF3D7_1324300	Moderate
-	conserved Plasmodium membrane protein, unknown function	PF3D7_1464500	Moderate
K Homology domain, type 1 superfamily	serine repeat antigen 8	PF3D7_0207300	Moderate
-	conserved Plasmodium protein, unknown function	PF3D7_0511400	Moderate
Aminotransferase, class I/classII	kelch domain-containing protein, putative	PF3D7_1205400	Moderate
-	conserved Plasmodium protein, unknown function	PF3D7_1223500	Moderate
-	gamete egress and sporozoite traversal protein, putative	PF3D7_1449000	Moderate
Anaphase-promoting complex subunit 1	conserved Plasmodium membrane protein, unknown function	PF3D7_0728100	Moderate
WD40 repeat	WD repeat-containing protein, putative	PF3D7_1004200	Moderate
Winged helix-like DNA-binding domain superfamily	conserved Plasmodium protein, unknown function	PF3D7_1145800	Moderate
Zinc finger, UBR-type	asparagine and aspartate rich protein 1	PF3D7_1233600	Moderate
Endonuclease/exonuclease/phosphatase superfamily	CCR4 domain-containing protein 1, putative	PF3D7_0519500	Moderate
-	conserved Plasmodium protein, unknown function	PF3D7_0826000	Moderate
-	conserved Plasmodium protein, unknown function	PF3D7_0316200	Moderate
B-block binding subunit of TFIIIC	conserved Plasmodium protein, unknown function	PF3D7_0403400	Moderate
-	conserved Plasmodium membrane protein, unknown function	PF3D7_0404600	Moderate
MORN	MORN repeat protein, putative	PF3D7_0511300	Moderate
TRAPP_II_complex_Trs120	conserved Plasmodium membrane protein, unknown function	PF3D7_0719900	Moderate
Anaphase-promoting complex subunit 1	cation transporting P-ATPase	PF3D7_0504000	Moderate
-	heptatricopeptide repeat-containing protein, putative	PF3D7_0608300	Moderate
Winged helix-like DNA-binding domain superfamily	tubulin--tyrosine ligase, putative	PF3D7_1147200	Moderate

Zinc finger, UBR-type	antigen 332, DBL-like protein	PF3D7_1149000	Moderate
-	cysteine repeat modular protein 3	PF3D7_1208200	Moderate
Schizont-infected cell agglutination, C-terminal domain	surface-associated interspersed protein 14.1 (SURFIN 14.1)	PF3D7_1477600	Moderate
-	Plasmodium exported protein (hyp1), unknown function	PF3D7_0301600	Moderate
Zinc finger C2H2-type	MMS19-like protein, putative	PF3D7_0505000	Moderate
CCR4-Not complex component, Not1	NOT family protein, putative	PF3D7_1417200	Moderate
-	liver specific protein 1, putative	PF3D7_1418100	Moderate

S5 Table. Non-synonymous mutations for the PfS47 gene.

Cluster	Collection Site	Code	Year	PfS47					
				68	194	236	242	247	248
Bv1 type	Peruvian Amazon Basin	CDR20014	2014	T	P	I	L	V	L
Bv1 type	Peruvian North Coast	CDR20001	2010	T	H	I	L	V	L
Bv1 type	Peruvian North Coast	CDR20002	2011	T	H	I	L	V	L
Bv1 type	Peruvian North Coast	CDR20003	2010	T	H	I	L	V	L
Bv1 type	Peruvian North Coast	CDR20005	2011	T	H	I	L	V	L
Clonet D	Peruvian Amazon Basin	CDR20012	2010	M	H	T	S	V	I
Acre-Loreto	Peruvian Amazon Basin	CDR20013	2010	T	H	T	S	V	L
Clonet D	Peruvian Amazon Basin	CDR20011	2007	M	H	I	L	V	L
Bv1 type	Peruvian Amazon Basin	CDR20010	2006	T	H	I	L	V	L
Clonet D	Peruvian Amazon Basin	MDP2017	2009	M	P	I	L	V	L
Clonet D	Peruvian Amazon Basin	MDP2018	2009	M	H	I	L	V	L
Acre-Loreto	Peruvian Amazon Basin	MDP3015	2010	T	H	T	S	V	I
Bv1 type	Peruvian Amazon Basin	MDP3120	2011	T	P	T	S	V	L
Bv1 type	Peruvian Amazon Basin	MDP3130	2011	T	H	I	L	V	L
Bv1 type	Peruvian Amazon Basin	MDP3207	2012	T	P	I	L	V	L
Bv1 type	Peruvian Amazon Basin	MDP3823	2014	T	H	I	L	V	L
Bv1 type	Peruvian Amazon Basin	MDP3860	2015	T	H	I	L	V	I
Bv1 type	Peruvian Amazon Basin	MDP3895	2015	T	P	T	S	V	I
Bv1 type	Peruvian Amazon Basin	MDP3962	2016	T	P	T	S	V	I
Bv1 type	Peruvian Amazon Basin	MDP3988	2016	T	H	I	L	V	L
Bv1 type	Peruvian Amazon Basin	MDP5048	2013	T	H	I	L	V	L
Bv1 type	Peruvian Amazon Basin	MDP6123	2017	T	P	T	S	V	I
Bv1 type	Peruvian North Coast	CDR20008	2010	T	H	I	L	V	L
Bv1 type	Peruvian North Coast	CDR20009	2010	T	P	I	L	V	I
Africa	Sudan	ERR1015544		T	H	T	S	V	I
Africa	Unknow	ERR1015568		T	H	T	S	V	I
Africa	Unknow	ERR1015546		T	H	T	S	V	I
Africa	Togo	ERR1015543		T	H	T	S	V	L
Africa	Africa (NF54)	SRR530501		T	P	T	S	V	I
Africa	Brazil (7G8)	SRR530164		T	H	I	L	V	L
Asia	Asia (MRA1241)	ALQ44019		T	H	I	S	V	L

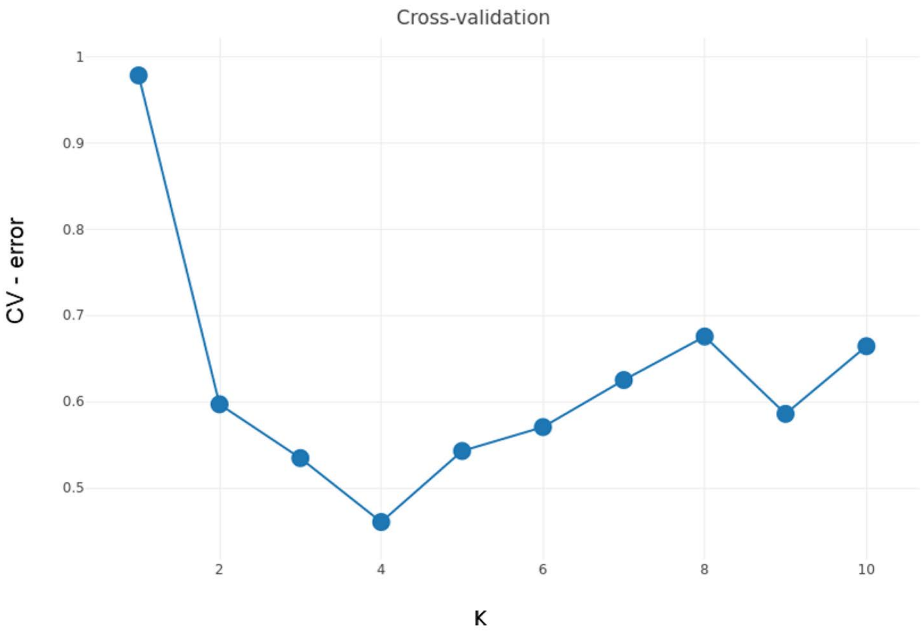

S1 Fig. Plot of Admixture cross-validation analysis from K=1 through k=10.

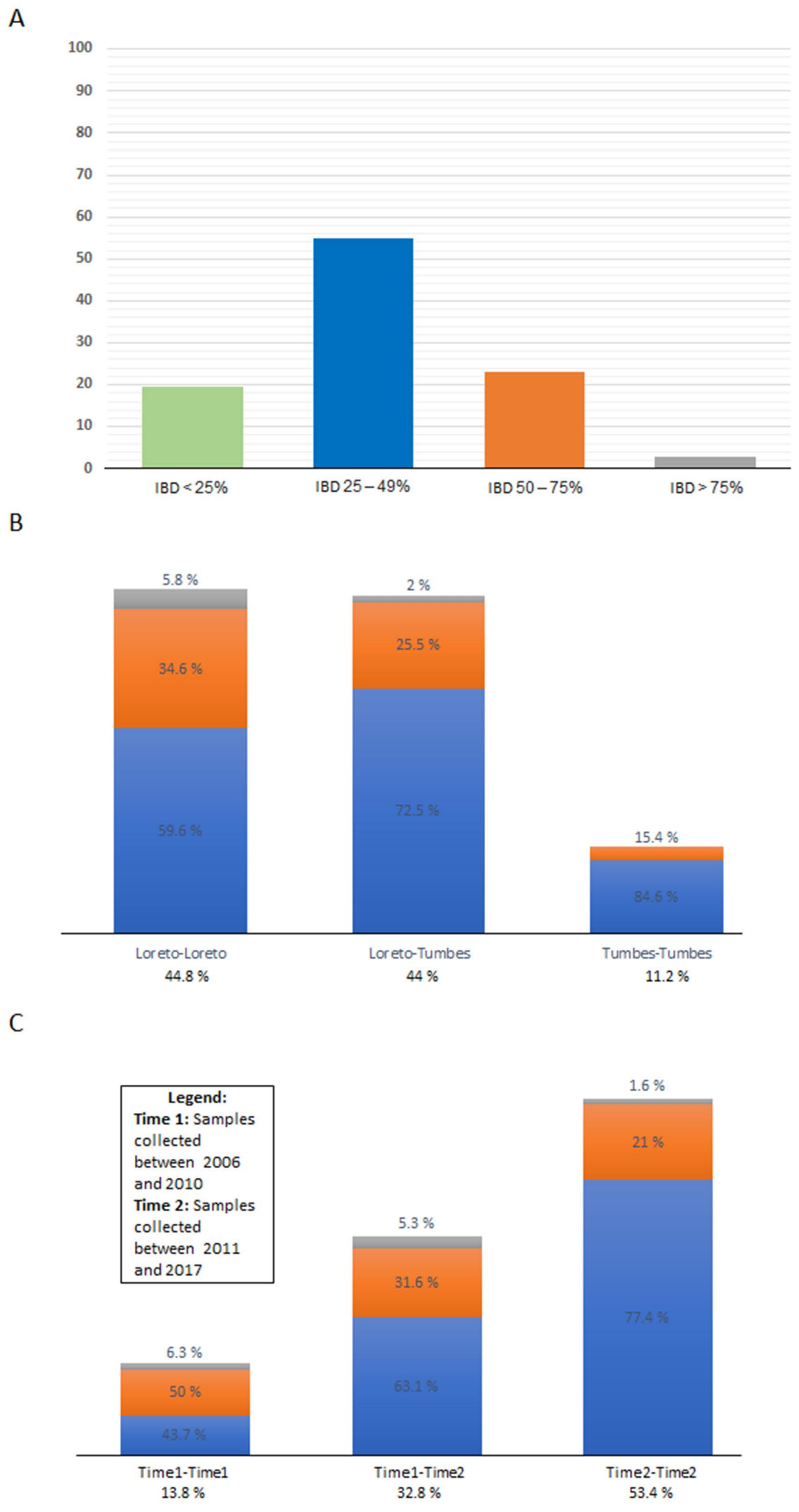

S2 Fig. Fractions of highly-related sample pairs partitioned in space and time. All data is showed in percent. Relatedness threshold used was more than 25% of the genome in IBD. A) Data sub-classified according to the fraction of the genome in IBD. Green shows low-relatedness (IBD <25%); blue, high-relatedness (25-49%); orange, very high-relatedness (50-75%) and gray, clonal samples (>75%). B) Fractions of related sample pairs partitioned by space. The data was compared as follows: Loreto vs. Loreto, Loreto vs. Tumbes and Tumbes vs. Tumbes. Colors represent the same data presented in figure 3A. C) Fractions of related sample pairs partitioned in time. The data were compared as follow: Time 1 vs. Time 1, Time 1 vs. Time 2 and Time 2 vs. Time 2. Time 1 represents samples collected between 2006 and 2010 and Time 2 samples collected between 2011 and 2017. Colors represent the same data presented in 3A.

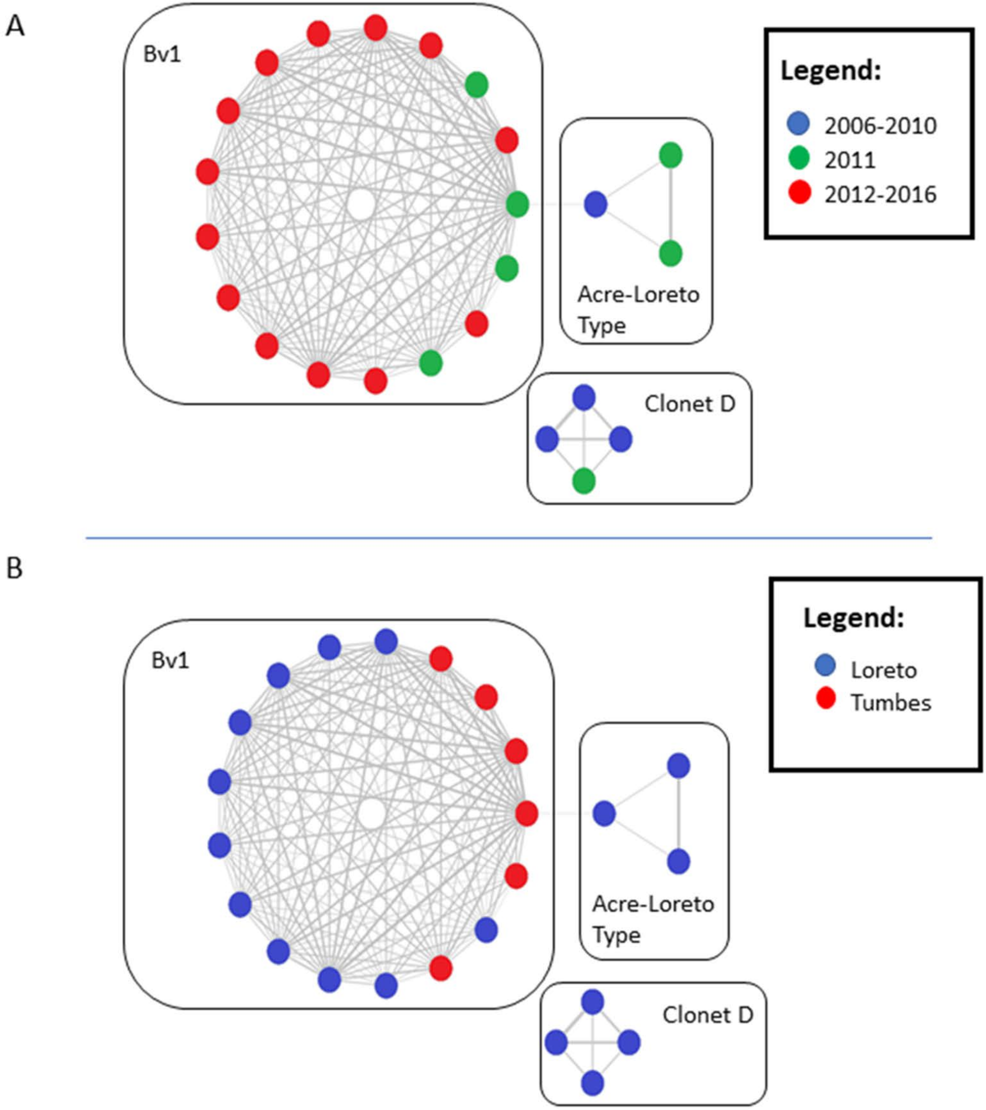

S3 Fig. Samples are showed with vertices and the average relatedness in proportional edge. Edge without relatedness was not plotted. A) IBD partitioned by time. Colors represented the sample collection time; blue since 2006 until 2009, green in 2011 and red since 2012 until 2017. B) IBD partitioned by collection site. Colors represented the sample collection site; Loreto in blue and Tumbes in red.

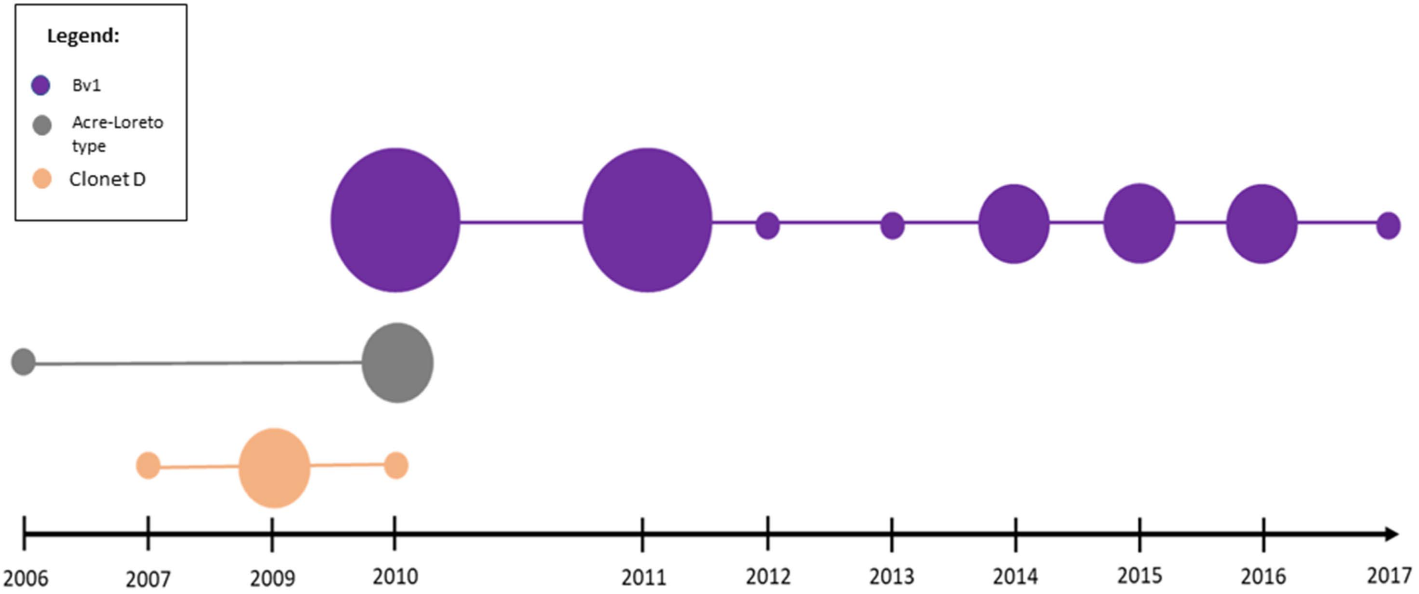

S4 Fig. Clonal components found during the time study. Clonal samples are showed in vertices which are proportional to the number of samples collected per year. Purple represent Bv1 type lineage, gray Acre-Loreto type and orange for Clonet D. The graphic shows an expansion of Bv1 since 2011.

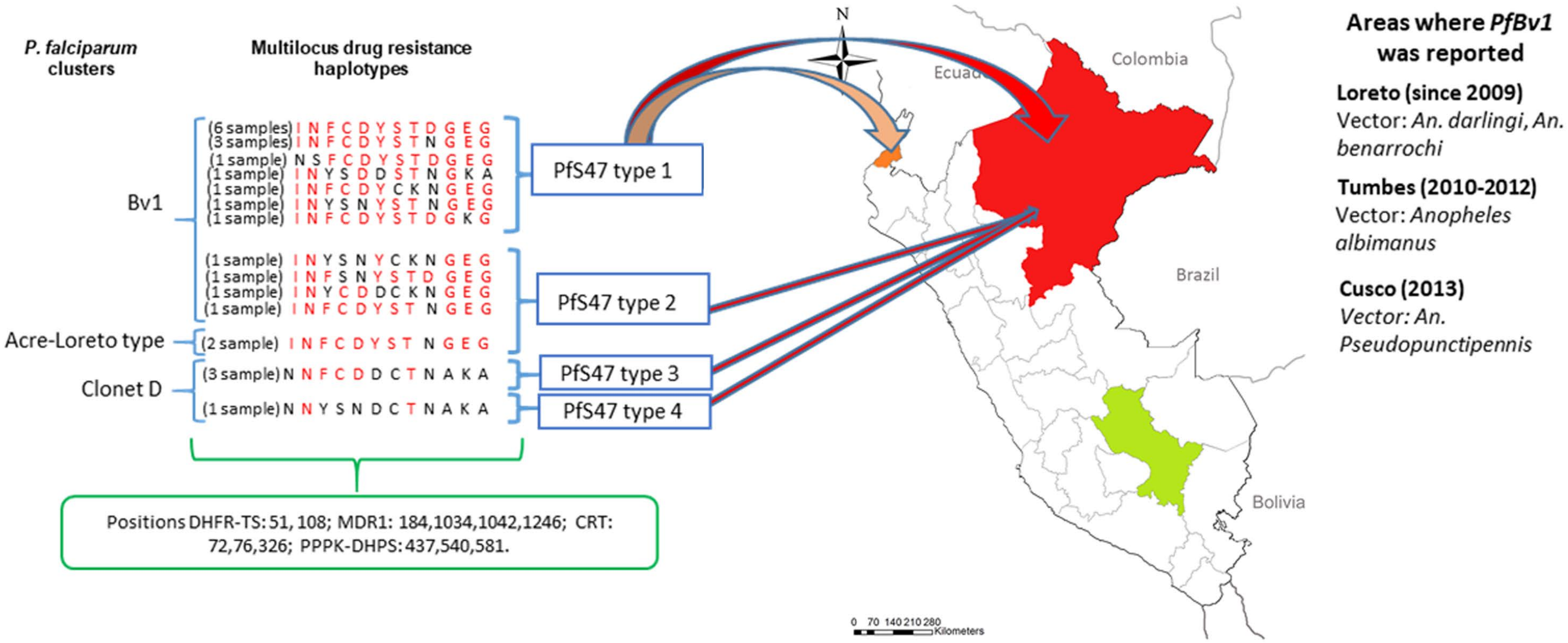

S5 Fig. Distribution of Peruvian circulating clusters of P. falciparum and the main vectors from each area. The map shows the Loreto region in the Northern Peruvian Amazon (red), the North Coast (orange) and the Cusco region of the recent Bv1 outbreak (green). Seven variants of Bv1 with PfS47 type 1 were distributed in Tumbes (North Coast) and Loreto (Amazon Basin) whereas the PfS47 type 2, 3 and 4 were restricted to Loreto. The outbreak reported in Cusco did not assessed the PfS47 type. The positions of the multilocus drug resistance haplotypes are described on the green chart. The map was created using ArcGIS online (ESRI Inc. Redlands, CA, USA. https://www.esri.com/) using open data obtained from GADM database of Global Administrative Areas, version 3.6. URL: www.gadm.org.
